# A novel modality for real-time measurement of provider happiness

**DOI:** 10.1093/jamiaopen/ooac009

**Published:** 2022-02-24

**Authors:** Narath Carlile, Sarah Tantillo, Michael Brown, David W Bates, Niteesh K Choudhry

**Affiliations:** 1 Internal Medicine, Brigham and Women’s Hospital, Boston, Massachusetts, USA; 2 Harvard Medical School, Boston, Massachusetts, USA

**Keywords:** burnout, quality of healthcare, measurement, healthcare provider

## Abstract

**Objective:**

Physician burnout is at epidemic proportions, impacts clinical outcomes, and is very costly. Although there is emerging data about effective interventions, most physicians at risk of burnout do not seek help. Survey-based measures exist which can quantify burnout within populations, but these are usually only administered episodically. We hypothesized that a novel modality for real-time measurement of happiness and stressors would be acceptable, scalable, and could provide new actionable insights.

**Materials:**

We developed a novel informatics system consisting of a networked smart button device, server, and analytics for measuring happiness, and stressors in real-time during clinical work. We performed an observational cohort study in 3 primary care clinics. Random and fixed effects modeling was used to analyze predictors of stress and happiness and we conducted a survey of usability and user acceptance of the novel system.

**Results:**

We captured 455 recordings across 392 provider days from 14 primary care providers. In total, 85% of users found the device easy to use, and 87% would recommend the system to their colleagues. Happiness and stressors were observed in all working hours of the day, with a 22% reduction in feeling (the proportion of happiness to stressors) across a clinical day.

**Discussion:**

We tested a novel system which providers found easy to use and enabled collection of detailed data. Limitations included being an observational study within a small number of clinics. A simple, unintrusive, scalable informatics system capable of measuring happiness, and stressors in real-time could be useful to healthcare organizations and teams.

## Lay Summary

Burnout is a maladaptive response to chronic stressors. Physician burnout is at epidemic proportions, impacts clinical outcomes, and is very costly. Survey-based measures exist which can quantify burnout within populations, but as yet we do not have real-time, scalable, system-wide signals (of stressors or satisfiers) which would allow organizations to implement and monitor interventions that might mitigate the development of burnout. We developed a novel informatics system consisting of a networked smart button device, server, and analytics for measuring happiness and stressors in real-time during clinical work. We tested this system in an observational study with doctors, nurse practitioners, and physician assistants in 3 different primary care clinics in our organization. Most of the users found the device easy to use and would recommend it to their colleagues. Two important implications of the study are that the system was acceptable to users and can be easily scaled allowing organizations to understand and evaluate the impact of wellbeing interventions, workflow and technology changes more quickly; and second that the detailed real-time data provide the opportunity to detect and address stress and happiness occurring during clinical work in new ways and allows for the possibility of proactive outreach. Limitations of the study include having a small number of providers in a limited number of clinics. A novel, scalable system capable of measuring happiness and stressors in real-time could be useful to healthcare organizations and teams in tackling the epidemic of burnout.

## BACKGROUND AND SIGNIFICANCE

Physician and team member burnout have reached epidemic proportions within many healthcare organizations with rates exceeding 50% in some settings.^1^ Burnout is “a work-related syndrome involving emotional exhaustion, depersonalization and a sense of reduced personal accomplishment.”^1^ Burnout is driven by work-related stressors including excessive workload, inefficient work processes such as documentation burden, perceived lack of input or control, and work/home conflict.^1^^,^^2^ Burnout impacts clinical outcomes, errors, productivity, absenteeism, and results in increased staff turnover.^3^^,^^4^ The annual estimated cost of burnout in the United States has been estimated to be $4.6 billion. Turnover costs alone are estimated to be $500,000 to 1 million per physician.^5^

Although there is emerging data about effective interventions to address burnout,^6^^,^^7^ most stressed physicians do not adequately address their own well-being or seek help.^8^ Proactively identifying physicians in need is not currently done in healthcare in a systematic way, with current approaches primarily being self-referral, or referral by supervisor. Survey-based measures are used to quantify the level and domains of burnout,^9^ and are widely used but these lack detail about when the stressors occur, are subject to reporting biases, and are usually only administered episodically.

A complementary approach would be to leverage near real-time systems that provide continuous data on stressors or satisfiers for healthcare workers. Existing digital systems for ecological momentary assessment have been studied within mental health^10^ and have been found to be feasible and well accepted, but had drawbacks such as participant fatigue with questionnaires and ethical concerns. Approaches to tracking and monitoring mood with passive digital data (such as step counts, call logs, sleep data, and heart rate data) do show correlations with perceived stress^11^ but have privacy concerns. Other measures such as salivary cortisol^12^ and heart rate variability^13^ have also been studied and seem to measure physiological states but have limitations regarding their association with stress and are more invasive, or require prolonged monitoring.

A tool to allow organizations to systematically identify stressors or satisfiers during work may overcome these limitations. This would allow a direct recording of actual perceived stressors (or satisfiers) and could directly identify when individual or groups of physicians experience persistent, ongoing stressors or if relative stress was increasing or decreasing as a result of interventions or changes (such as workflow redesign). To our knowledge, no such tool has been studied in healthcare.

## OBJECTIVE

In this study, we used a novel tool to measure the happiness and stressors for providers in real time. We hypothesized that this would be acceptable to providers and might offer insights into the causes of stress and happiness for clinical teams. This could provide a modality that could scale across organizations and could be used in the future to provide predictive analytics that would allow a more nuanced, and perhaps automated early intervention to prevent burnout from developing or progressing.

## MATERIALS AND METHODS

### Informatics system

**Figure 1.**
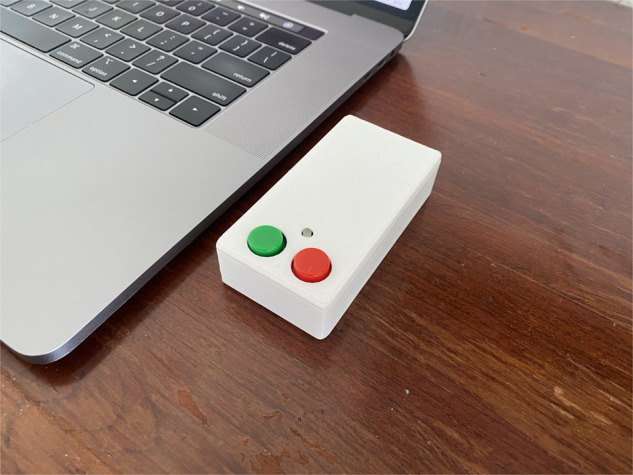
The BE HAPPI device.

We developed a unique informatics system that includes a self-contained device consisting of 2 buttons—1 indicating the user was feeling happy, and another indicating that the user was feeling stressed. These buttons are connected to a microprocessor that sends the signal from the button to a server via an encrypted channel through a wireless network. The device has a unique identifier which is linked to a specific provider and location. The devices are inexpensive and can be connected to any available network through an embedded configuration portal. The server records the date and time the click was received as well as the device identifier. Automated individual and aggregate group reports can be generated by the system on an as-needed basis. The device was designed and built by the primary investigator and his daughter, with design input from the entire research team (Figure 1).

### Overall study design

We conducted an observational cohort study among primary care providers to study the usability of the devices in clinical environments. The study was approved by the Mass General Brigham IRB. We enrolled practices within our primary care network representing different clinical environments, specifically a community health clinic, a large academic teaching practice, and a private practice. Clinic directors were contacted about the study and invited to participate. Providers at practices agreeing to participate were given a presentation outlining the study aims as well as the use of the device. At the conclusion of the presentation, all clinical providers were invited to participate.

### Study procedures

Providers completed a study consent and an initial questionnaire and then received their devices. If they worked from home as well, they also received a device for home. They were instructed to press the green button when they felt happy during clinical work and to press the red button if they felt stressed. They were asked to log data only during clinical activities. The study participants received a weekly report of their data. Each clinic received an aggregate report of data at the end of the study. At the end of the study, the providers completed a final questionnaire.

### Statistical considerations

R version 4.1.2 by the R Foundation for Statistical Computing was used for the analysis.

Since we had repeat samples from each user, we used random and fixed effects modeling to obtain the estimates and *P* values for the analysis of overall feeling. A normalized overall feeling score from 0 to 100 was calculated for each hour based on the proportion of happy to stressed observations. In this scale, 0 would be always stressed, 50 is neutral, and 100 would be always happy.

To understand significant predictors of stress and happiness, we used fixed and random effects logistic regression modeling, using a correction for multiple hypotheses so that a *P* value of .0167 (0.05/3) would be considered significant. Results are presented as odds ratios with associated confidence intervals. The predictors included hour of day, day of week, gender, role, clinic type, and location (home vs clinic). Since nurse practitioners (NPs) and physician assistants (PAs) were not present at every site Role was collapsed to “MD” (medical doctor) and “NP or PA” categories.

Usability and user acceptance of the devices were captured in the final study survey. A final presentation of aggregate results was presented to all clinic participants where open-ended qualitative feedback was captured.

## RESULTS

**Figure 2.**
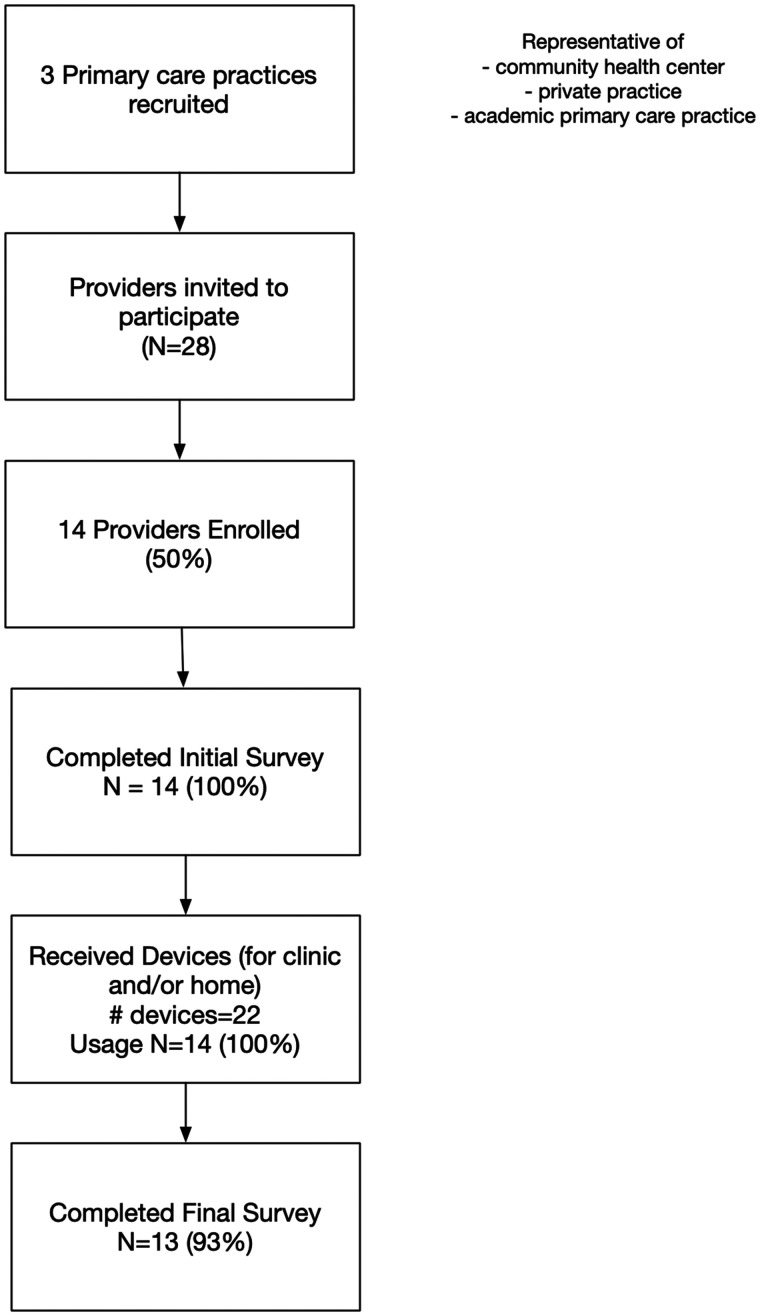
Consort diagram of clinic enrollment.

We obtained 455 measures of feeling using the smart buttons across 392 provider days from 14 primary care providers from October 2020 to December 2020 (Figure 2), with a mean of 1.08 measures per user per day (with a range of 0–26). Subjects within the study were primarily female physicians working in a community health center. Table 1 shows the baseline characteristics of the study participants.

**Table 1.** Provider demographics (and data recorded)

	Subjects	Observations, total (mean per subject)	Happy observations, total (mean per subject)	Stressed observations, total (mean per subject)	Feeling per hour, mean (CI)
All	14	455 (33)	253 (18)	202 (14)	54% (41 to 65)
Provider types					
MD	9	301 (33)	173 (19)	128 (14)	57% (39 to 74)
NP	2	62 (31)	31 (16)	31 (16)	50% (−61 to 100)
PA	3	92 (31)	49 (16)	43 (14)	47% (−9 to 100)
Gender					
Female	12	344 (29)	198 (17)	146 (12)	57% (42 to 69)
Male	2	111 (56)	55 (28)	56 (28)	42% (−166 to 100)
Clinic types					
Academic practice	3	102 (34)	60 (20)	42 (14)	58% (6 to 100)
Private practice	4	71 (18)	24 (6)	47 (12)	38% (2 to 73)
Community health center	7	282 (40)	169 (24)	113 (16)	61% (45 to 77)
Location					
Clinic	13	354 (27)	200 (15)	154 (12)	54% (42 to 67)
	5	101 (20)	53 (11)	48 (10	54% (30 to 78)

Among these subjects, 85% of users found the device easy to use, 77% said they would like to continue using the device, 87% would recommend the use of the device for their colleagues, 54% of users would also have liked to use an app version, 15% a watch application, 8% a desktop application, and 23% a texting modality.

**Figure 3.**
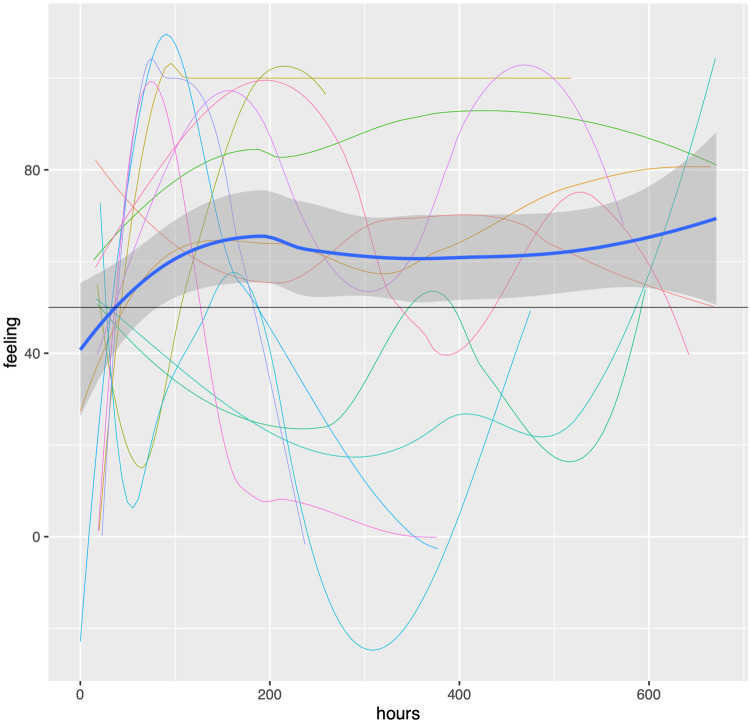
Feeling over time with individual estimation curves (thin lines) and central tendency (blue line). The black reference line indicates a feeling of 50% which is neutral. The lines are estimates of central tendency for individuals and so can extend below 0 and above 100.

Figure 3 displays the estimated normalized feeling for individual providers as well as the overall estimated feeling over the course of the study. Feelings tended to be neutral and trended up slowly over time, but this was not a statistically significant change (effect = 0.02% change per hour, CI −0.009 to 0.041). The detail of the data made available through the system revealed significant heterogeneity for individual providers.

**Figure 4.**
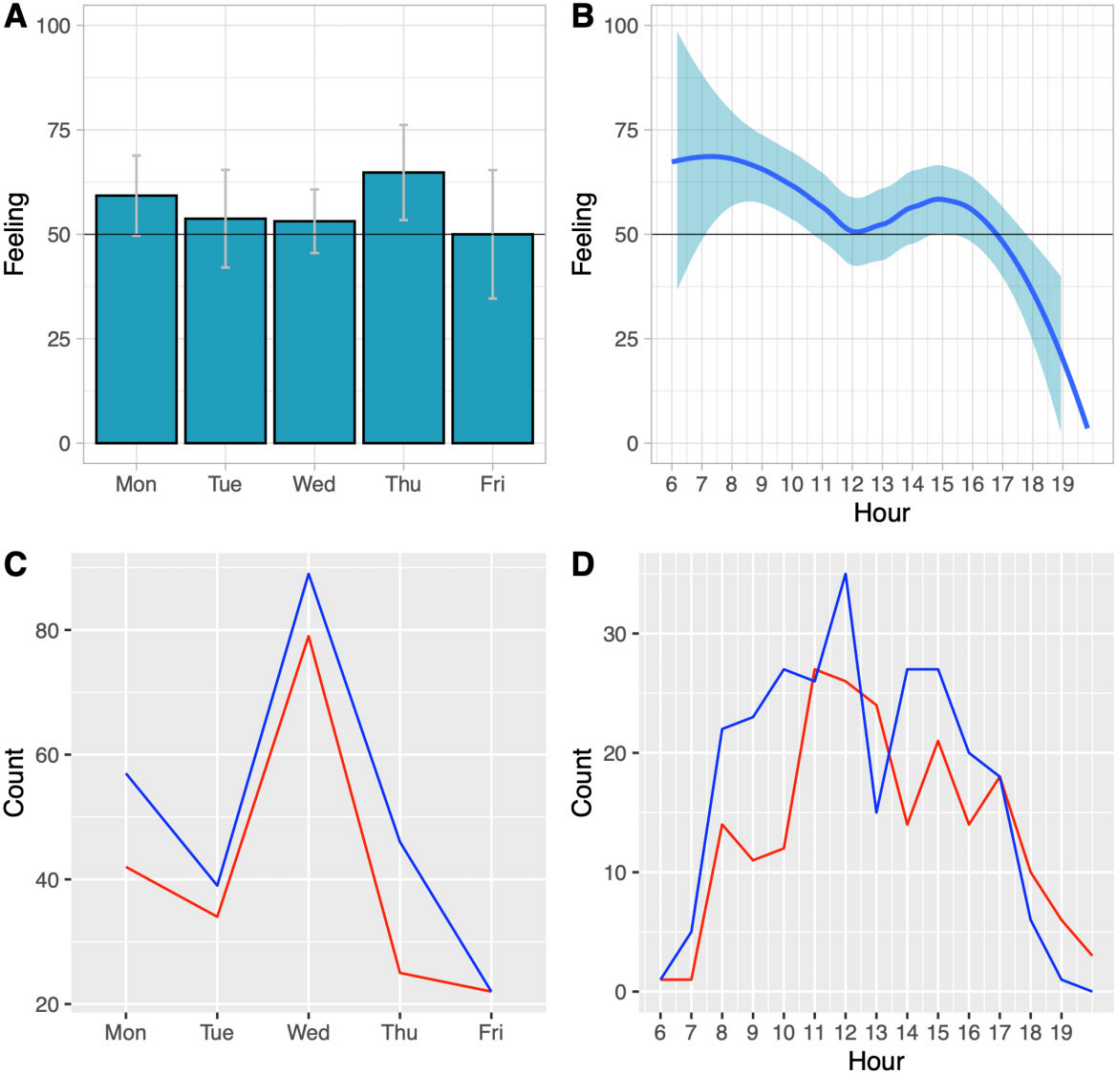
(A) The average feeling by day of week. (B) The average feeling by hour of day. (C) The number of happy recordings (in blue) and stress (in red) by day of week, and (D) this by hour of day.

Figure 4 shows graphs of the overall feeling per day of the week and hour of the day, and also shows the counts for happy and stressed observations at those times. Happiness and stressors were observed on all days and working hours, but the proportionate relationship varied. There was a decrease in the recorded feeling throughout the day, and changes across days of the week, with Monday and Thursday having a higher proportion of happiness.

Results of analysis of the multivariate predictors of feeling are shown in Table 2. This analysis showed that recorded feeling decreased by 2.2% each hour across the day. Over a 10-hour working day^14^ this would be a reduction of 22%. Wednesday and Friday had lower happiness with odds ratios of being less happy on those days compared to Monday of 0.5 and 0.4, respectively but after correction for multiple analyses this was not thought to be significant within the current data set. The only univariate results which were significant were increases in stress by hour of day, and clinic type.

**Table 2.** Predictors of feeling per hour, stress, and happiness

	% Feeling (CI)	*P*	OR stressed (CI)	*P*	OR happy (CI)	*P*
(Intercept)	95.5 (66.8 to 124.2)		0.028 (0.08–0.99)		14.5 (4.28–49.4)	
Hour	−2.21 (−3.78 to −0.6)	.006*	1.07 (1.00–1.15)	.064	0.897 (0.833–0.966)	.004*
Tuesday vs Monday	−3.11 (−19.7 to 13.5)	.714	0.92 (0.44–1.93)	.830	0.607 (0.281–1.31)	.201
Wednesday vs Monday	0.04 (−13.4 to 13.5)	.995	0.81 (0.44–1.48)	.493	0.498 (0.267–0.927)	.0280
Thursday vs Monday	6.82 (−8.84 to 22.5)	.394	0.46 (0.22–0.98)	.043	0.748 (0.354–1.58)	.448
Friday vs Monday	−4.18 (−22.7 to 14.4)	.659	1.02 (0.43–2.41)	.946	0.410 (0.171–0.983)	.046
Gender: male (compared with Female)	−13.7 (−45.7 to 18.4)	.425	2.60 (0.77–8.80)	.124	0.537 (0.209–1.38)	.198
Role: NP or PA (compared with MD)	−12.0 (−36.6 to 12.5)	.360	2.19 (0.87–5.53)	.095	0.522 (0.250, 1.09)	.084
Private practice (compared with community health center)	−20.08 (−45.3 to 5.2)	.151	2.4 (0.88–6.49)	.084	0.420 (0.191–0.924)	.031
Academic center (compared with community health center)	−0.098 (−28.3 to 28.1)	.995	1.09 (0.38–3.10)	.878	1.40 (0.612–3.21)	.425
Home (compared with clinic)	4.65 (−11.9 to 21.2)	.583	0.63 (0.31–1.28)	.205	1.17 (0.583–2.35)	.657

*A *P* value of less than .0167 is considered significant (given multiple hypotheses tested).

NP: nurse practitioners; PA: physician assistants.

## DISCUSSION

We developed and tested a novel system that allows for real-time assessment of provider stressors and happiness and provided weekly individual, as well as group feedback. The devices were easy to deploy and were available in clinic and in other practice locations such as homes where virtual care is being provided. In qualitative feedback, providers shared that using the device helped a number of them to do quick “life-hacks” that let them quickly decrease their stressors and improve their happiness at work, such as bringing a full meal on longer days in clinic, or realizing they were arriving at work already stressed (and so focused on relaxation techniques at home). The devices were also reported to provide some catharsis in having their stress “recognized” and helped some providers to recognize that they had more happiness at work than they had realized. In qualitative feedback from group meetings, the time of day, and day of week patterns triggered a number of ideas for possible improvements including establishing a “blocked” wrap-up session at the end of the day among providers that would help them complete their sessions on time. Medical directors expressed their interest in using this to measure whether systemic changes (driven externally such as electronic medical record (EMR) or policy changes, or internally such as clinic wide wellness initiatives) had positive or negative effects across their teams, and to use this data to make adjustments in their clinics (such as the blocked end-of-day wrap-up session described above) as well as using it to more effectively advocate for systemic changes with organizational leadership.

Burnout poses a tremendous problem in many industries, but particularly in healthcare.^3^ Not only does it increase turnover, which is costly, but it also adversely impacts clinical outcomes, increases errors, decreases productivity, and increases absenteeism.^3^ It is likely that this has worsened during the Coronavirus pandemic.^15^ Current methods to measure burnout^16^^,^^17^ have value, but generally rely on self-administered scales which can take time from busy clinicians. This can make it possible for these instruments to worsen stressors or make it less likely for those who are most stressed to complete them. They also require infrastructure to distribute and gather and analyze responses and are intended to quantify rates of burnout in populations rather than to identify individual providers who may be burned out or at risk of burnout. As currently implemented, these surveys do not provide individual providers or groups with timely and comprehensive data that they can use to make ongoing adjustments to address chronic and emerging stressors. In short, physician burnout is an important problem in medicine, current ways to measure burnout have limitations, and there are currently no systematic ways to identify clinicians who are facing persistent stressors and so may be at risk of burnout. A “biomarker” of persistent stress, a “burnout troponin” could be a very useful additional tool to address this epidemic.

We found that the devices were used an average of 33 times per individual over the 28-day study period and were perceived as easy to use by 85% of the users. Remarkably, 77% of the users were interested in continuing to use the devices beyond the study duration. We also found a statistically significant trend of decreasing overall feeling (a proportion of happiness to stress) as a function of the hour of day, with a decline of 22% across a 10-hour clinical day. Predictors of decreased happiness or increased stress may also include some days of the week and practice type, which provide possible opportunities for focused improvements in the future.

Our study has 2 important implications. First, it demonstrated that a system that offers the ability to monitor stress and happiness in real-time at scale across organizations is acceptable and will be used by providers. Since it can be deployed at scale to all team members it has the potential to help measure short- and long-term impacts of wellness and wellbeing interventions holistically across teams and organizations. This could be very important in helping organizations more quickly understand the impact of workflow, technology, and wellbeing interventions on all their teams.

Second, the granularity and timeliness of the data available from this system, and the heterogeneity it revealed, provide the opportunity to detect and address stress and happiness occurring during clinical work in new ways. The data from this initial deployment showed information that was of interest and could be acted on by both individual clinicians (who focused on individual factors that they could quickly affect) as well as organizational leaders (who focused on the group level data showing more system-level factors). In addition, since the data is available in real-time it also allows for the possibility of providing proactive outreach with evidence-based interventions to help prevent persistent stressors from developing into burnout and supporting and strengthening factors that contribute to happiness during clinical work.

This study has a number of limitations. First, it had a small number of mostly female subjects within a limited number of clinics. These individuals did provide a reasonable number of observations and we used fixed and random effects modeling to account for the correlation of the observations. The study was likely not sufficiently powered to be able to detect differences among some demographic groups such as gender differences. A second limitation was that the devices used did not capture other measures that have been considered of interest in studies of physiological stress such as heart rate variability, galvanic skin response, or salivary cortisol. Although these may be interesting correlates, they can be more invasive, and providers had expressed concern regarding continuous monitoring during our study enrollment so we expect that more invasive or continuous monitoring that was not user initiated, would likely limit scale and adoption. The action of deciding to press the stress or happy button may also present more direct data regarding the person’s perceived emotional state, and so may in fact be a more accurate measure of emotion than these other measures.

In addition to full-time clinicians, many providers practice part-time, and may have their first clinic not on Monday but on another day of the week. Future work could include analysis of stressors and satisfiers related to specific session periods in addition to the days of the week and time of the day.

An additional limitation in this study was that we did not seek or ask the provider to provide detailed information about activities causing stress or happiness. In this study, we focused on understanding the acceptability of the device, and on some of the generic predictors of stressors and happiness in clinicians and observing their individual and team responses to their own happiness and stress graphs. In future work, these happiness and stress reactions could be mapped to EMR log data to identify activities that could be affecting clinicians, or our texting bot or an app could prompt the user after button presses to journal about the activity causing that reaction. Additionally, the server technology could be used with embedded happy and stress buttons within the EMR or other hospital systems. This more detailed data could be very valuable to healthcare organizations when implementing EMR, or workflow changes. Even with the current system that does not track specific activity data, happiness and stressor data being generated across an organization could provide important information to understand the effectiveness, or limitations of wellness and wellbeing interventions, of widespread technology changes (such as EMR changes) or the impact of pandemics and other crises on healthcare staff.

Finally, another limitation of this work is that it did not yet address whether acting on the signals created by the system can help to mitigate burnout. This was designed as a usability and acceptability study of the devices, and the data created by the system. Working with our organization the team created a user-centered interventions list that could be proactively activated by thresholds from this signaling system. The effect of this overall system (signal plus intervention and monitoring) is planned for future study.

## CONCLUSION

We developed and deployed a novel modality for real-time measurement of provider happiness and stressors. This system was easy to deploy and was found to be easy to use by providers, the majority of whom would recommend this system to their colleagues. In addition to the usability data, the detailed data revealed new insights into patterns of stress and happiness across groups of clinicians. Future work should include wider deployment to additional organization types and geographies, deployment to all team members within the healthcare organization, as well as more detailed activity tracking through EMR logs, embedding in the EMR, or through digital journaling. The effect of adding proactive outreach to providers experiencing greater stress and decreased happiness should also be tested. We hope that simple, unobtrusive systems like this will be useful in helping to bring more nuanced, real-time, and holistic measures to healthcare teams and organizations as they apply systemic wellness and wellbeing interventions to address the chronic stressors that are contributing to this worsening epidemic of healthcare worker burnout.

## FUNDING

This study was funded by the Brigham Care Redesign Incubator Startup (BCRISP) program of the Brigham and Women’s Physician Organization.

## AUTHOR CONTRIBUTIONS

NC conceived of the study, designed and developed the hardware and software, conducted the study, designed, and completed the analysis of the data, and drafted and revised the article. SAT assisted with the design of the visualizations and the study, coordinated the study, assisted in the literature review, and revised the article. MB assisted with the design of the devices, the visualizations, and study design, and revised the article. DWB assisted with the conception and design of the devices, visualization, and study, was the executive sponsor for the study, collaborated on the design of the study and data collection tools, and data analysis, and drafted and revised the article. NKC assisted with the conception and design of the devices, visualization, and study, collaborated on the design of the study, data collection tools, collaborated on the design, and execution of the data analysis, and drafted and revised the article.

## References

[1] West CP, Dyrbye LN, Shanafelt TD. Physician burnout: contributors, consequences and solutions. J Intern Med 2018; 283 (6): 516–29. 29505159 10.1111/joim.12752

[2] *Factors Affecting Clinician Well-Being and Resilience – Conceptual Model – Clinician Well-Being Knowledge Hub*. https://nam.edu/clinicianwellbeing/resources/factors-affecting-clinician-well-being-and-resilience-conceptual-model/. Accessed May 13, 2021

[3] Han S, Shanafelt TD, Sinsky CA, et al Estimating the attributable cost of physician burnout in the United States. Ann Intern Med 2019; 170 (11): 784–90. 31132791 10.7326/M18-1422

[4] Dyrbye LN, Shanafelt TD, Sinsky CA, *et al*. Burnout among health care professionals: A call to explore and address this underrecognized threat to safe, high-quality care. *NAM Perspectives*. Discussion Paper. Washington, DC: National Academy of Medicine; 2017. https://doi.org/10.31478/201707b.

[5] Organizational Cost of Physician Burnout. https://edhub.ama-assn.org/steps-forward/interactive/16830405. Accessed May 28, 2021.

[6] Dyrbye LN, Shanafelt TD, Gill PR, Satele DV, West CP. Effect of a professional coaching intervention on the well-being and distress of physicians: a pilot randomized clinical trial. JAMA Intern Med 2019; 179 (10): 1406. 31380892 10.1001/jamainternmed.2019.2425 PMC6686971

[7] Patel RS, Sekhri S, Bhimanadham NN, Imran S, Hossain S. A review on strategies to manage physician burnout. Cureus 2019; 11 (6)e4805. 31404361 10.7759/cureus.4805 PMC6682395

[8] Fridner A, Belkić K, Marini M, Gustafsson Sendén M, Schenck-Gustafsson K. Why don't academic physicians seek needed professional help for psychological distress?. *Swiss Med Wkly* 2012; 142: w13626. 10.4414/smw.2012.13626 22802214

[9] Valid and Reliable Survey Instruments to Measure Burnout, Well-Being, and Other Work-Related Dimensions. National Academy of Medicine. https://nam.edu/valid-reliable-survey-instruments-measure-burnout-well-work-related-dimensions/. Accessed May 13, 2021.

[10] Sedano-Capdevila A, Porras-Segovia A, Bello HJ, Baca-García E, Barrigon ML. Use of ecological momentary assessment to study suicidal thoughts and behavior: a systematic review. Curr Psychiatry Rep 2021; 23 (7): 41. 34003405 10.1007/s11920-021-01255-7

[11] Acikmese Y, Alptekin SE. Prediction of stress levels with LSTM and passive mobile sensors. Proc Comput Sci 2019; 159: 658–67.

[12] Hellhammer DH, Wüst S, Kudielka BM. Salivary cortisol as a biomarker in stress research. Psychoneuroendocrinology 2009; 34 (2): 163–71. 19095358 10.1016/j.psyneuen.2008.10.026

[13] Kim H-G, Cheon E-J, Bai D-S, Lee YH, Koo B-H. Stress and heart rate variability: a meta-analysis and review of the literature. Psychiatry Investig 2018; 15 (3): 235–45. 10.30773/pi.2017.08.17 PMC5900369 29486547

[14] How many hours are in the average physician workweek? American Medical Association. https://www.ama-assn.org/practice-management/physician-health/how-many-hours-are-average-physician-workweek. Accessed May 6, 2021.

[15] Wahlster S, Sharma M, Lewis AK, et al The COVID-19 pandemic’s impact on critical care resources and providers: a global survey. Chest 2020; 159 (2): 619–33. 32926870 10.1016/j.chest.2020.09.070 PMC7484703

[16] Maslach C, Leiter MP. Early predictors of job burnout and engagement. J Appl Psychol 2008; 93 (3): 498–512. 18457483 10.1037/0021-9010.93.3.498

[17] Dolan ED, Mohr D, Lempa M, et al Using a single item to measure burnout in primary care staff: a psychometric evaluation. J Gen Intern Med 2015; 30 (5): 582–7. 25451989 10.1007/s11606-014-3112-6 PMC4395610

